# Human Psychological Disorder towards Cryptography: True Random Number Generator from EEG of Schizophrenics and Its Application in Block Encryption's Substitution Box

**DOI:** 10.1155/2022/2532497

**Published:** 2022-06-21

**Authors:** Muhammad Fahad Khan, Khalid Saleem, Mohammad Mazyad Hazzazi, Mohammed Alotaibi, Piyush Kumar Shukla, Muhammad Aqeel, Seda Arslan Tuncer

**Affiliations:** ^1^Department of Computer Science, Quaid-i-Azam University, Islamabad, Pakistan; ^2^Department of Software Engineering, Foundation University Islamabad, Islamabad, Pakistan; ^3^Department of Mathematics, College of Science, King Khalid University, Abha, Saudi Arabia; ^4^Department of Management Information Systems, College of Business Administration, University of Tabuk, Tabuk, Saudi Arabia; ^5^Department of Computer Science & Engineering, University Institute of Technology, Rajiv Gandhi Proudyogiki Vishwavidyalaya, Bhopal, Madhya Pradesh, India; ^6^Department of Psychology, Foundation University Islamabad, Islamabad, Pakistan; ^7^Department of Software Engineering, Firat University Faculty of Engineering, Elazig, Turkey

## Abstract

Schizophrenia is a multifaceted chronic psychiatric disorder that affects the way a human thinks, feels, and behaves. Inevitably, natural randomness exists in the psychological perception of schizophrenic patients, which is our primary source of inspiration for this research because true randomness is the indubitably ultimate valuable resource for symmetric cryptography. Famous information theorist Claude Shannon gave two desirable properties that a strong encryption algorithm should have, which are confusion and diffusion in his fundamental article on the theoretical foundations of cryptography. Block encryption strength against various cryptanalysis attacks is purely dependent on its confusion property, which is gained through the confusion component. In the literature, chaos and algebraic techniques are extensively used to design the confusion component. Chaos- and algebraic-based techniques provide favorable features for the design of the confusion component; however, researchers have also identified potential attacks on these techniques. Instead of existing schemes, we introduce a novel methodology to construct cryptographic confusion component from the natural randomness, which are existing in the psychological perception of the schizophrenic patients, and as a result, cryptanalysis of chaos and algebraic techniques are not applicable on our proposed technique. The psychological perception of the brain regions was captured through the electroencephalogram (EEG) readings during the sensory task. The proposed design passed all the standard evaluation criteria and validation tests of the confusion component and the random number generators. One million true random bits are assessed through the NIST statistical test suite, and the results proved that the psychological perception of schizophrenic patients is a good source of true randomness. Furthermore, the proposed confusion component attains better or equal cryptographic strength as compared to state-of-the-art techniques (2020 to 2021). To the best of our knowledge, this nature of research is performed for the first time, in which psychiatric disorder is utilized for the design of information security primitive. This research opens up new avenues in cryptographic primitive design through the fusion of computing, neuroscience, and mathematics.

## 1. Introduction

Schizophrenia is a multifaceted psychiatric disorder, which consists of several varied causes such as environmental, developmental, and genetic factors. Due to numerous complications of its causes, inevitable natural randomness exists in the electroencephalographic readings of patient's psychological responses. Patients who suffer from schizophrenia show randomness in their clinical presentation of symptoms, characteristics, and related prognosis. It is distinguished by three major clusters of symptoms consisting of cognitive symptoms including impairment of short- or long-term working memory, negative symptoms like social withdrawal, and positive symptoms like hallucinations or delusions. These symptoms stimulate diverse neural activities in the different regions of brain. Natural randomness has been acknowledged as the ideal method for cryptography and a lot of researchers endorse the true random numbers for cryptography due to the reason that true random numbers are irreversible, unpredictable, and unreproducible, even if their internal construction and response history are identifiable to the adversaries [1–8].

Naturally, in the characteristics of the schizophrenic patients, diverse spectrum of disorders inevitably exists, which was our core source of inspiration because these disorders are the potential source of natural randomness. For example, in the delusion characteristic, patients lose their brain control due to their delusionary beliefs about the world around them. The loss of control stimulates uncertain and indistinct neural activities in different parts of the brain. These delusions could include grandiose, erotomaniac, and persecutory. Another characteristic of schizophrenic patients is the variation in the presentation of their sensory hallucinations, which differs between each patient. These hallucinations could be auditory, visual, tactile, gustatory, or olfactory. These hallucinations are also responsible for the arbitrariness of neural activities in brain regions. The third characteristic is a derailment, in which patients have variations in the thinking patterns and these disorganized thinking patterns are also a cause of irregular neural activity in different brain regions. The last characteristic is grossly disorganized or catatonic behavior, which causes variation in their presentation of motor behavior due to the imbalanced neural activities. These involuntary motor behaviors can range from childlike “silliness” to unpredictable agitation, which causes difficulty in goal-directed behavior.

Protecting secret information is a global challenge, and block cipher has been a standout among the most reliable option by which security is accomplished [9–12]. Block ciphers belong to the family of deterministic algorithms that operate on the fixed length of bits (*n*), called a block. A block cipher algorithm divides the plaintext into several fixed-length blocks of *n* bits, to produce a block of ciphertext of *k* bits. Block cipher combines both confusion and diffusion components within a round function and repeats the function multiple times to produce a ciphered text. Advanced Encryption Standard and Data Encryption Standard are the most prominent block ciphers. For the block ciphers, differential and linear attacks are considered very powerful attacks [13–17]. The main objective of the differential attack is to find the nonrandom pattern of the output, and for this objective, the attacker attempts to impose a certain set of input to track the differences in the output. Similarly, the main objective of the linear attack is to try to learn the linear association between the parity bits of cipher text, plaintext, and the symmetric key. Responsibility to make the correlation between ciphertext and the key, as undetectable as possible, is only on the confusion component, as well as resistance against the cryptanalysis attacks totally depends upon the confusion component [13–22]. The confusion component of the block cipher is normally known as substitution box (S-box) or nonlinear block cipher primitive. Nonlinear block cipher primitive transforms *m* bits input to *n* bits output by using S: {0,1}^*n*^⟶{0,1}^*k*^.

The ultimate goal of this research is to propose a methodology for the problem “how to construct the nonlinear primitive of block cipher using the strength of true randomness.” The core concept of this research is to extract true random bits, by calculating the difference between each electrode reading of one patient and those of all other patients, and to design a technique for the generation of nonlinear primitive of block cipher. The remaining study is arranged as follows: Section 2 presents our main contribution; Section 3 describes attacks on existing confusion component designs; Section 4 explains the proposed scheme; Section 5 presents the results and its evaluation; and Section 6 presents the application of the proposed dynamic confusion components in image encryption technique.

## 2. Contribution

The main contribution of this research is as follows:

- A novel method is proposed, to generate true random bits from the psychological perception of schizophrenic patients. As test, one million true random bits are assessed through the NIST statistical test suite, and the results proved that the psychological perception of schizophrenic patients is outstanding source of true randomness.
- Instead of algebraic structures and chaotic systems, our technique relies on inevitable natural randomness, which are existing in EEG of schizophrenic patients for the design of confusion component, and as a result, attacks of algebraic- and chaos-based techniques are not applicable and irrelevant for our proposed technique.
- To the best of our knowledge, this nature of research is performed for the first time, in which psychiatric disorder is utilized for the design of any block cipher primitive.
- This research opens up new avenues in cryptographic primitive design through the fusion of computing, neuroscience, and mathematic.
- As the application of our proposed dynamic confusion components, an image cipher based on confusion-diffusion principal is also developed and the resultant encrypted images are examined through various security analyses and statistical tests. All the results of these tests are passed, and it also confirms that the proposed confusion components are competent enough for the image cipher.

## 3. Attacks on Confusion Component Design Schemes

As mentioned earlier, chaos- and algebraic-based techniques are extensively used to design the confusion component. Chaos- and algebraic-based techniques provide favorable features for the design of confusion components; however, researchers have also identified various cryptanalysis on these techniques including interpolation attacks [9–12], Gröbner basis attack [13–19], SAT solver [20–27], linear and differential attacks [28–42], XL attacks [43–45], and XSL attack [9, 46–55]. Similarly, chaos-based techniques are also commonly applied in the designs of confusion components [56–68], dynamical degradation of chaotic systems [69–73], predictability [74–85], discontinuity in chaotic sequences [70, 86–90], small number of control parameters [76, 77, 91, 92], finite precision effect [70–72, 86, 88], and short quantity of randomness [71, 72, 86, 88–90, 93–96].

On the other side, a lot of researchers endorse the true random numbers for cryptography due to the purpose that true random numbers are unpredictable, unreproducible, and irreversible, even if their inner structure and past responses are known to the adversary. [1–8]. Our proposed technique extracts true random bits, from the readings of patient's electrode scalp sites (Fz, FCz, Cz, FC3, FC4, C3, C4, CP3, CP4) during the sensory task.

## 4. Proposed Design

**Figure 1:**
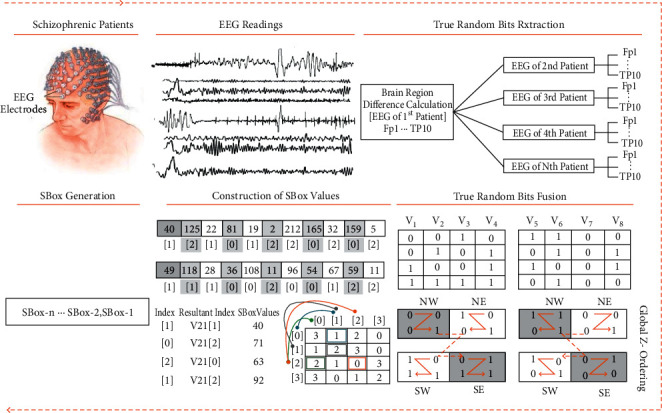
Proposed system design.

The proposed technique has two phases: true random bits extraction and dynamic generation of confusion components. The system architecture diagram is depicted in Figure 1 and the whole design is explained in the following phases.

**Phase 1 .**

True random bits extraction

**Figure 2:**
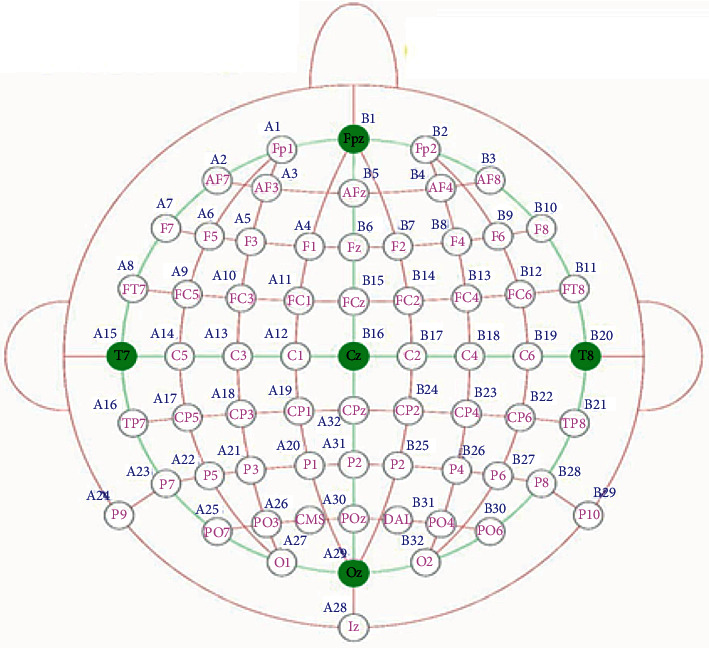
64-channel active-electrode layout.

- Acquire EEG readings from the basic sensory button press task
- The dataset that is used in this research was obtained from Refs. [97, 98], and for this, forty-nine schizophrenia patients were selected by professional and clinical psychologists after the initial screening of schizophrenia symptoms. Symptoms of the schizophrenia are assessed through three standardized psychological instruments: Scale for Negative Symptoms (SANS), Scale for Positive Symptoms (SAPS), and Positive and Negative Syndrome Scale (PANSS). The age range of the schizophrenia patients is 20 to 60 (*μ* = 42.82, *σ* = 13.12) years, and different subtypes of schizophrenic patients included such as residual schizophrenia, paranoid schizophrenia, undifferentiated schizophrenia, schizophrenia unknown subtype, schizoaffective disorder, and disorganized schizophrenia. Event-related potential (ERP) averages of nine electrode scalp sites (Fz, FCz, Cz, FC3, FC4, C3, C4, CP3, CP4) are obtained, and readings of the electroencephalography are continuously digitalized at 1024 Hz. The topological positions of the 64-channel, active-electrode layout is illustrated in Figure 2 [98]. The sensory task given to the participants consisted of a button press at every 1–2 seconds, to deliver 1000 Hz, 80 dB sound pressure level, and tones with zero delay between press and tone onset. The task was stopped after 100 tones had been delivered.
- Difference calculation between each electrode reading of one patient and each electrode reading of all other patients
- Each reading of the 1st channel is subtracted, from the 1st channel reading, of all other patients. Similarly, each reading of the 2nd channel is subtracted, from the 2nd channel reading of all other patients. Subtracted readings of every channel are stored individually in vector data structure and then parsed into binary format. This process is repeated over the readings of 64 channels and 4900 vectors generated. As test, one million of these binary bits are assessed through the NIST statistical test suite, and the results of Table 1 proved that the psychological perception of schizophrenic patients is a good source of true randomness.
- True Random Bits Fusion

**Figure 3:**
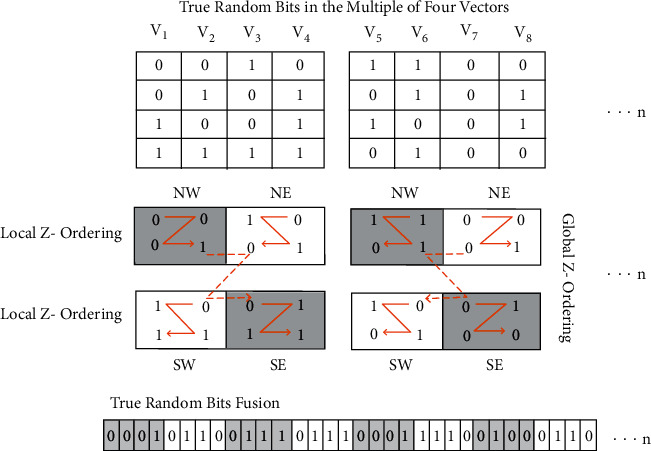
True random bits fusion.

The output of the last step is fused through the proposed DIFFERENCE_FUSION () algorithm, which is attached in annexed (Figure S1). A visual representation of the algorithm is depicted in Figure 3. This algorithm takes true random bits in the multiple of four vectors and then traverse in a specific order based on z-ordering. If the value of quadrant NW is 0, then retrieve bits from left to right, and if the value of quadrant NW is 1, then retrieve bits from right to left. Two variations of the z-ordering scheme are implemented here: the first is local *z,* which operates on 2 × 2 bits, and the second is global *z,* which operates on 2 × 2 local *z*.

**Phase 2 .**

Dynamic generation of confusion components

**Figure 4:**
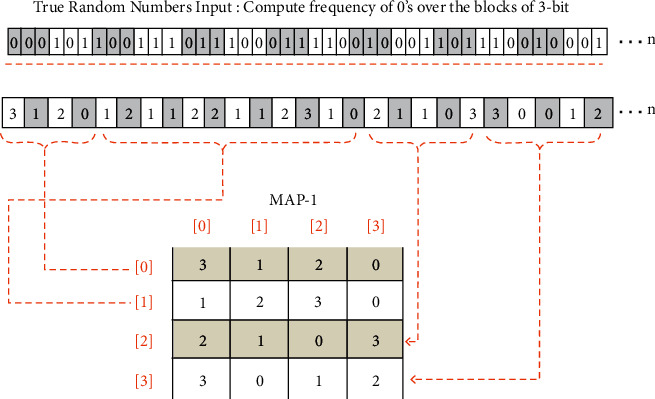
Difference-based two-dimensional map generation.

- Difference-based Two-Dimensional Map Generation (D2DMG)
- Vectors of the last step are passed as parameters to the D2DMG() algorithm for the generation of two-dimensional maps. Visual representation of the algorithm is depicted in Figure 4, and the D2DMG() algorithm is attached in annexed (Figure S2).
- Dynamic Confusion Component Generator (DCCG)

**Figure 5:**
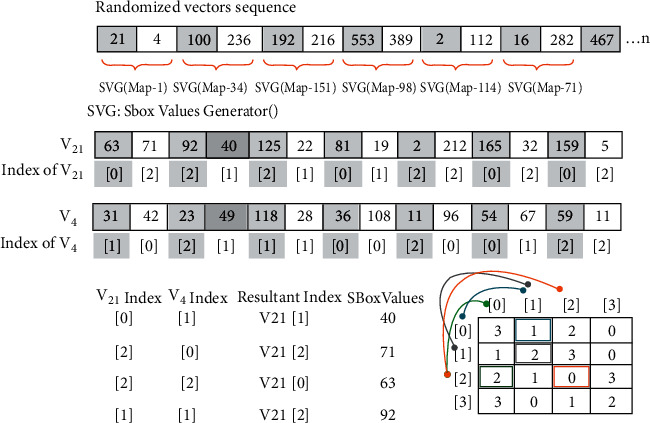
Confusion value generator.

Pairwise randomly traverse all vectors from Phase 1 and then assign arbitrary indexes. Arbitrary indexes are produced simply by applying the module 3 operation on every byte of the vector. Here, arbitrary indexes work as indexes of the vector elements. To get the values of the confusion component, parameters (pair of vectors with their arbitrary index and map with its index) are passed to the ConfusionValuesGenerator() algorithm. ConfusionValuesGenerator algorithm is attached in annexed (Figure S3), and the visual representation of the algorithm is depicted in Figure 5. Due to the pure randomized nature, on every call, this algorithm returns 0 to 8 values. Resultant stream of the ConfusionValuesGenerator( ) algorithm was passed to the DCCG() algorithm for the generation of dynamic confusion components. The DCCG algorithm returns dynamic confusion components depending upon the size of stream; the DCCG algorithm is attached in annexed (Figure S4). From the results, six confusion components are randomly picked as samples, and first randomly picked confusion component and its inverse is shown in Tables 2 and 3 respectively, and the remaining five confusion components are shown in annexed (Table S1). The reverse S-box algorithm is shown in Algorithm 1.

## 5. Results Evaluation

In this section, sample confusion components of Section 4 are evaluated through the standard confusion component evaluation criteria [32–44], which includes bit independence criterion(BIC), linear approximation probability (LP), strict avalanche criterion (SAC), nonlinearity score, and differential approximation probability (DP).

### 5.1. Nonlinearity

Nonlinearity is one of the most important confusion component properties, which indicates the resistance ability of confusion components against the linear attacks, and the nonlinearity of cipher is expressed by the nonlinearity score. It is known as the smallest distance of Boolean function from the set of affine functions. The nonlinearity score is the total number of bits altered to get the nearest affine function in the Boolean truth table. To calculate the nonlinearity score, the distance of all affine functions and Boolean function is determined. When the initial distance is calculated, the nearest affine function is achieved by changing the amount of bit values in the Boolean function's truth table. The Walsh spectrum defines the nonlinearity of a Boolean function by using the following formula:

(1) $$\begin{array}{c} N_{g}=2^{n-1}\left(1-2^{-n}\underset{\varphi \varepsilon \text{GF}\left(2^{n}\right)}{\text{max}}\left|S\left(g\right)\left(\varphi \right)\right|\right), \end{array}$$

where  *S*_(*g*)_(*φ*) is defined as

(2) $$\begin{array}{c} S_{\left(g\right)}\left(\varphi \right)=\sum_{\varphi \in \text{GF}\left(2^{n}\right)}{\left(-1\right)}^{g\left(x\right)⊕x.\varphi }, \end{array}$$

where *φ* is a n-bit vector and *φ* ∈ GF(2^*n*^). The dot product between *x* and *φ* is denoted as *x* · *φ*:

(3) $$\begin{array}{c} x\cdot \varphi =x1⊕\varphi 1+x2⊕\varphi 2\cdots +xn⊕\varphi n. \end{array}$$

The nonlinearity score of our randomly picked confusion components 1,2,3,4,5,6 is 110.50, 106.75, 106.50, 106.75, 107.50, and 107.25, respectively. In Table 4 we can see that the nonlinearity score of our proposed confusion components is higher or equal from the state-of-the-art techniques(year 2020 to 2021).

### 5.2. Strict Avalanche Criteria (SAC)

SAC specify that all the output bits will be modified with 1/2 probability by flipping a bit of input. SAC analyze the impact of avalanche effects in encryption. The change in the input generates a number of changes in the output. Having an even output pattern prevents linear attacks. Therefore, the changes in the output bits must be independent. SAC counts the number of changed output bits caused by complementing a single bit of input. All output bits will deviate with the probability of one half for an algorithm to be more secure. To test the SAC of the confusion component, we used the dependency matrix. S-box fulfils the SAC property, if all the elements and mean value in the dependency matrix are approximately equal to 0.5. The offsets of the dependence matrix are calculated by the following formula:

(4) $$\begin{array}{c} S\left(g\right)=\;\frac{1}{n^{2}}\;\sum_{1\leq r\leq n}\sum_{1\leq w\leq n}\left|\frac{1}{2}-Qr,w\left(g\right)\right|, \end{array}$$

where

(5) $$\begin{array}{c} Qr,\;w\left(g\right)=2^{-n}\sum_{x\varepsilon B^{n}}gw\left(x\right)⊕gw\left(x⊕e_{r}\right), \end{array}$$

*e*_*r*_=[*θr*, 1*θr*, 2 … *θr*, *n*]^*T*^ is the transpose of matrix *θ*_*r*,*w*_=0, *r* ≠ *w* Or *θ*_r,w_*θ*_*r*,*w*_=1, *r*=*w*

The SAC (average) score of our randomly picked six confusion components (1,2,3,4,5,6) is 0.498779, 0.500244, 0.503662, 0.497314, 0.500732, and 0.508545, respectively. These results proved that our proposed confusion components are enough capable. The SAC result of confusion component-1 presented in Table 5 is the sample

### 5.3. BIT Independent Criterion (BIC)

BIC is used to analyze the output bits behavior by changing the input bits. Confusion component holds the BIC property when output bits behave independently from each other. BIC characteristic states that output bits *j* and *k* will modify individually if any single input bit *i* is reversed. This will improve the proficiency of confusion function. The independence between pair of avalanche variables is measured through the coefficient of correlation. The bit independence of the *j*^th^ and *k*^th^ bits of *B*^*ei*^ is

(6) $$\begin{array}{c} \text{BIC}\left(b_{j},b_{k}\right)=\underset{1\leq i\leq n}{\max}\left|\text{corr}\left(b_{j}^{ei},b_{j}^{ei\prime }\right)\right|. \end{array}$$

Shannon's confusion function(C) is represented as *C*: {0, 1}^*n*^ ⟶{0, 1}^*n*^. BIC parameter for Shannon's confusion function is measured by the given mathematical expression:

(7) $$\begin{array}{c} \text{BIC}\left(C\right)=\underset{1\leq j,k\leq n}{\max}\text{BIC}\left(b_{j},b_{k}\right). \end{array}$$

The shift in output bits is an important parameter for determining the strength of the encryption process. The average BIC score of our randomly picked confusion components from 1 to 6 is 0.50105, 0.50272, 0.50112, 0.50223, 0.50105, and 0.50105, respectively. These results proved that our proposed confusion components strongly fulfill the bit independent criteria. The SAC-BIC results of confusion component-1 presented in Table 6 are the sample.

### 5.4. Linear Approximation Probability (LP)

LP is another important criteria for evaluating Shannon's confusion component. LP is the function's capability to avoid linear attacks and is the highest value of the disparity of an event. The input bit's parity selected by the mask *γ*^1^ and the output bit's parity selected by the *γ*^2^ mask are equal. The masks of input and output bits are evaluated to obtain the imbalance of an event. Linear approximation probability is measured by the following mathematical expression:

(8) $$\begin{array}{c} {\text{LP}}_{f}={\text{max}}_{\gamma 1,\gamma 2\neq 0}\left|\frac{\left\{xɛX|x\cdot \gamma 1=S\left(x\right).\gamma 2\right\}}{2^{n}}-\frac{1}{2}\right|, \end{array}$$

where *γ*^1^ represents the input mask and *γ*^2^ represents the output mask in the above equation. *X* represents the set of all possible inputs, and 2^n^ is the total number of elements in the confusion component. The maximum LP score of our confusion components(1 to 6) is 0.1171875,0.1328125,0.12500, 0.1328125, 0.140625, and 0.140625, respectively; these results also fulfills the LP criteria.

### 5.5. Differential Approximation Probability (DP)

DP characteristic examines the XOR distribution among the input and output bits. In order to be resilient against the differential attacks, the XOR values of all outputs must have equal probability with the XOR values of all inputs. In the differential approximation table, the probability of all the XOR values of input and the probability of all XOR values of output are equal. The exclusive-or distribution among the inputs and outputs of S-box is calculated by

(9) $$\begin{array}{c} \text{DP }\left(\Delta w⟶\Delta z\right)=\left[\frac{\;\#\left\{\mathrm{wɛX}|S\left(w\right)⊕S\left(w⊕\Delta w\right)=\Delta z\right\}}{2^{i}}\right]. \end{array}$$

Here *X* represents the set of all possible input values and 2^i^ represents cardinality of set. The maximum DP score of our confusion components (1 to 6) is 0.046875, 0.046875, 0.046875, 0.054688, 0.039062, and 0.054688, respectively; here, we can see that these results also fulfills the DP criteria. As a sample, the DP results of the confusion component-1 are presented in Table 7.

## 6. Application of Proposed Dynamic Confusion Components in Image Encryption

**Figure 6:**
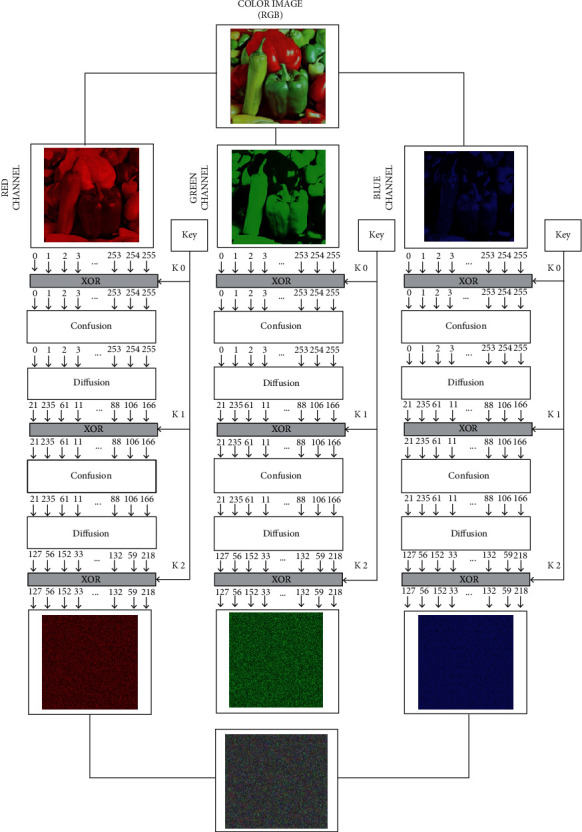
Confusion- and diffusion-based image cipher.

As the application of our proposed dynamic confusion components, an image cipher based on confusion-diffusion principal is developed, which is depicted in Figure 6. The structure of the mage cipher is depicted in Figure 6. It consists of repeating rounds of dynamic confusion layers, static diffusion layer, and the key addition, which make them hard for cryptanalysis. For the key generation process, the chaotic interval of the logistic map and tent map is enhanced by synthesizing the parameters of both maps to obtain the increased keyspace [86]. The chaotic field of the logistic map only lies in the range between 3.57 ≤ *σ* ≤ 4, and similarly, the chaotic field of the tent map lies in the range between 2 ≤ *σ* ≤ 4. Logistic map and tent map are defined in (10) and (11), respectively, and their enhanced chaotification structure of logistic tent system(LTS) is defined in (12). Finally for the subkey generation, divide the resultant values of LTS into the blocks of 256 bytes. In the same way for the permutation process, apply XOR operation on the values generated from (11) and (12). These resultant values are in the range between 0 and 255. Select first 256 distinct values as permutation. We examined the encrypted images through various security analyses and statistical tests including NPCR, UACI, correlation-coefficient analysis, and 2D, 3D histogram analysis. All the results of these tests are passed; it also confirms that the proposed confusion is competent enough for the image cipher:

(10) $$\begin{array}{c} z_{n+1}=\sigma z_{n}\left(1-z_{n}\right),\;0<\sigma \leq 4;\;z_{n}\in \left[0,1\right]. \end{array}$$

(11) $$\begin{array}{c} z_{n+1}=\left\{\begin{array}{c} \gamma \frac{z_{n}}{2}z_{i}<\frac{1}{2},\\ \frac{\gamma \left(1-z_{n}\right)}{2}z_{i}>\frac{1}{2}, \end{array}\right)\;0<\sigma \leq 4;\;z_{n}\in \left[0,1\right]. \end{array}$$

(12) $$\begin{array}{c} z_{n+1}=\left\{\frac{\left(\sigma z_{n}\left(1-z_{n}\right)+\left(4-\sigma \right)z_{n}/2\right)\text{ mod }255z_{i}<\left(1/2\right)}{\left(\sigma z_{n}\left(1-z_{n}\right)+\left(4-\sigma \right)\left(1-z_{n}\right)/2\right)\text{ mod }255z_{i}>\left(1/2\right)}.\right) \end{array}$$

### 6.1. Resistance against Differential Analysis

**Figure 7:**
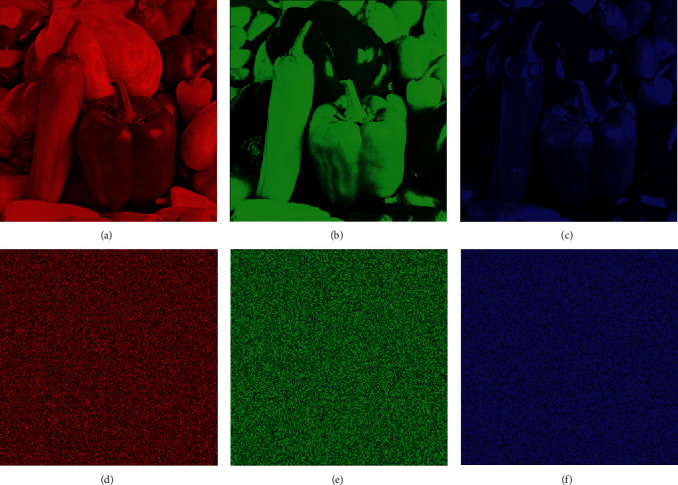
Original and encrypted test image of the pepper. (a) Before encryption(Channel:R); (b) before encryption (Channel:G); (c) before encryption (Channel:B); (d) after encryption(Channel:R); (e) after encryption (Channel:G); (f) after encryption (Channel:B).

The key requirement of the encryption algorithm is its ability to resist the differential attacks. Differential cryptanalysis is difficult when a small shift in original image will generate completely different ciphered image. We examined the image encryption results on various standard color test images (Lena, pepper, nature, bird, baboon, grapes, sparrow, butterfly), and here as a sample, original image pepper over the RGB channels is shown in Figures 7(a)–7(c) and their correspondent cipher pictures are presented in Figures 7(d), 7(e), and 7(f). The NPCR and UACI are the two frequently used tests of the image cipher to check the strength against the differential attacks. NPCR is defined as follows [124, 125]:

(13) $$\begin{array}{c} \text{NPCR}=\frac{\sum_{i,j}D\left(i,j\right)}{W\times H}\times 100\%. \end{array}$$

*D*_(*i*,*j*)_ is described as *D*_(*i*,*j*)_ = 0 if *I* (*i, j*) = *J*(*i, j*), D_(i,j)_ = 1 if *I*(*i, j*) = *J*(*i, j*)

UAIC measure the mean variation of pixel intensity of two encrypted images at same location. It is determined by

(14) $$\begin{array}{c} \text{UACI}=\frac{1}{W\times H}\times \left[\sum_{i,j}\times \frac{\left|C_{1}\left(i,j\right)-C_{2}\left(i,j\right)\right|}{255}\right]\times 100\%,\\ f\left(x\right)=\left\{\begin{array}{c} 0,\;\text{if }C_{1}\left(i,j\right)=C_{2}\left(i,j\right),\\ 1,\;\text{if }C_{1}\left(i,j\right)\neq C_{2}\left(i,j\right), \end{array}\;\right. \end{array}$$

where *C*1(*i*, *j*) and *C*2(*i*, *j*) indicate the pixel value of two encrypted images at location (*i*, *j*). W represents the number of rows and H presents the number of columns of the plain image. The encryption security is improved with a large UACI value. The NPCR and UACI are measured through the following formulas:

(15) $$\begin{array}{c} {\text{NPCR}}_{E}=\left(1-2^{-n}\right)\times 100\%,\\ {\text{UACI}}_{E}=\frac{1}{2^{2n}}\frac{\sum_{i=1}^{2^{n}-1}i\left(i+1\right)}{2^{n}-1}\times 100\%=\frac{1}{3}\left(1+2^{-n}\right)\times 100\%, \end{array}$$

where *n* is the number of bits used to denote the various bit planes of an image. High values of UACI and NCPR have strong resistance against differential attacks.  Table 8 indicates the values of NPCR and UACI. NPCR and UACI values of our encrypted images are near to 99.63 and 336.50, respectively, which are very good results.

### 6.2. Correlation Coefficient Analysis

**Figure 8:**
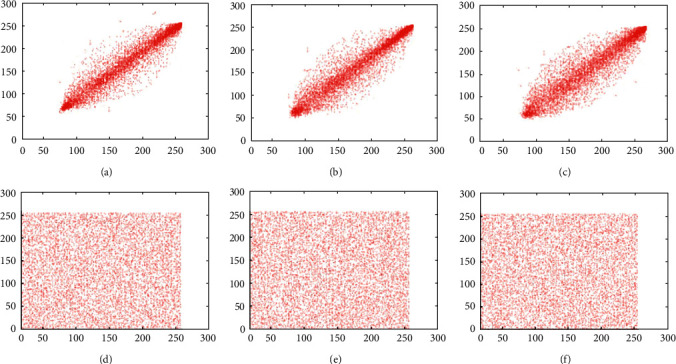
Scatter plots of the test image pepper over the R channel. (a) Plain image (direction: horizontal); (b) plain image (direction: vertical); (c) plain image (direction: diagonal); (d) cipher image (direction: horizontal); (e) cipher image (direction: vertical); (f) cipher image (direction: diagonal).

**Figure 9:**
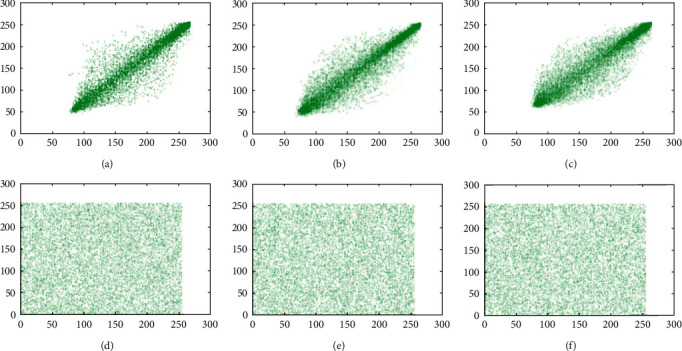
Scatter plots of the test image pepper over the G channel. (a) Plain image (direction: horizontal); (b) plain image (direction: vertical); (c) plain image (direction: diagonal); (d) cipher image (direction: horizontal); (e) cipher image (direction: vertical); (f) cipher image (direction: diagonal).

**Figure 10:**
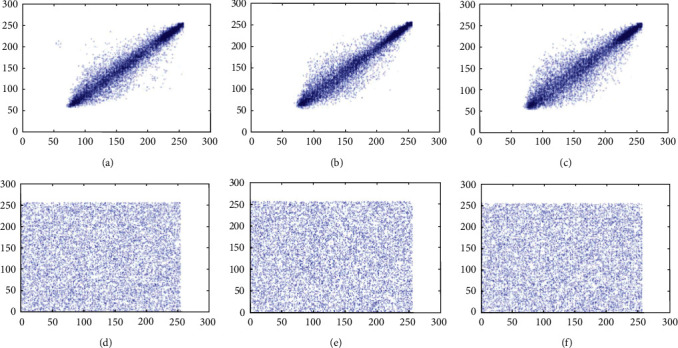
Scatter plots of the test image pepper over the B channel. (a) Plain image (direction: horizontal); (b) plain image (direction: vertical); (c) plain image (direction: diagonal); (d) cipher image (direction: horizontal); (e) cipher image (direction: vertical); (f) cipher image (direction: diagonal).

Neighbor pixels of the unencrypted images are extremely correlated and can show visual traits to the adversaries. An efficient cipher technique would reduce the correlation between adjacent pixels of an encrypted image in all the three directions. Before the encryption, the correlation coefficient value should be around 1 and after the encryption should be around 0. Adjacent pixel pairs of the test image pepper are plotted in Figures 8, 9 and 10. From the both original and encrypted images, 1000 pixels are plotted in the diagonal, horizontal, and vertical direction. Correlation coefficient among two neighboring pixels are calculated by

(16) $$\begin{array}{c} r_{xy}=\frac{\mathrm{cov}\left(x,y\right)}{\sqrt{D\left(x\right)}\sqrt{D\left(y\right)}},\\ \mathrm{cov}\left(x,y\right)=\frac{1}{N}\sum_{i=1}^{N}\left(x_{i}-E\left(x\right)\right)\left(y_{i}-E\left(y\right)\right),\\ D\left(x\right)=\frac{1}{N}\sum_{i=1}^{N}{\left(x_{i}-E\left(x\right)\right)}^{2},\\ E\left(x\right)=\frac{1}{N}\sum_{i=1}^{N}x_{i}, \end{array}$$

where *x*_*i*_ and *y*_*i*_ show the values of two adjacent pixels and N is the total number of duplets. The mean value of *x*_*i*_ is denoted by *E*(*x*), and the mean value of *y*_*i*_ is denoted by *E*(*y*). The calculated value of the correlation coefficient in plain images is closer to 1 along diagonal, horizontal, and vertical directions, whereas the value of correlation coefficient in encrypted image is closer to 0. We can see that the values of the correlation coefficient over the encrypted images are totally different from the values of plain images, so the correlation coefficient attack fails to provide any clue of the original image. The results of the correlation coefficient analysis on horizontal, vertical, and diagonal directions are displayed in Table 9.

### 6.3. Histogram Analysis

**Figure 11:**
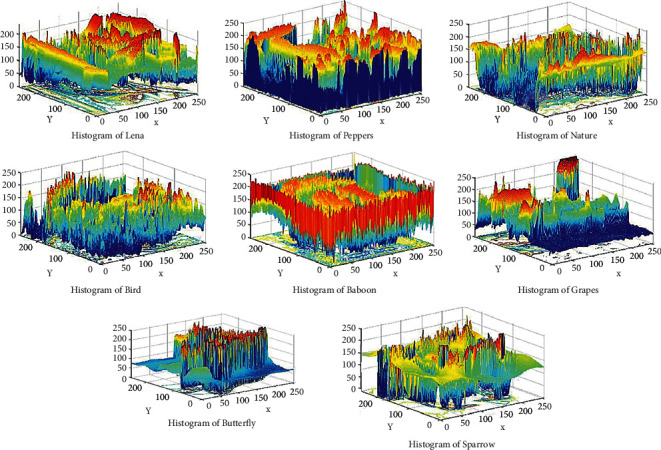
3D Histogram of the original images.

**Figure 12:**
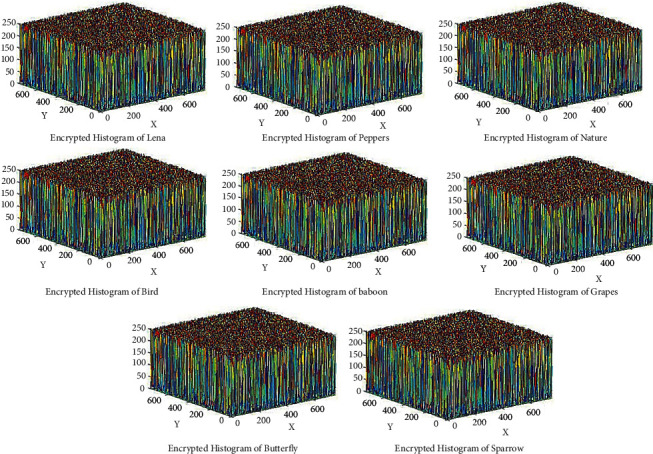
3D Histogram of the encrypted images.

The histogram is the graphical representation of the distribution of pixels in the picture by measuring a number of pixels at each intensity level. Analyzing the histogram shows how pixels are distributed over encrypted image. Effective cipher encrypts the original image into the cipher image, which contains random RGB pixel. In Figure 11, we can see that 3D histogram of the standard test images shows some information, but in Figure 12, encrypted test images have uniformly random pixel values. The histogram of the encrypted and original images are completely different, so the attacker cannot extract any relation between encrypted image and plain image.

## 7. Conclusion

Randomness is a fundamental feature in nature and a valuable resource for the cryptography. First time, this nature of research is performed in which psychiatric disorder is utilized for the generation of truly random bits, and based on these true random bits, confusion components are constructed. Instead of algebraic- and chaotic-based approaches, our technique relies on inevitable natural randomness, which exists in the EEG of schizophrenic patients, and as a result, attacks of chaos- and algebraic-based techniques are bypassed in our proposed approach. For the evaluation of the true random bits, NIST statistical test suite was adopted, and for the evaluation of the confusion component, standard evaluation criteria were adopted. As a test case, one million true random bits are assessed through the NIST statistical test suite, and the results proved that the psychological perception of schizophrenic patients is a good source of true randomness. Confusion components are evaluated through SAC, LP, DP, BIC, and nonlinearity. The outcomes of these criteria verified that the proposed confusion component is effective for block ciphers. We will expand this research in future, for the dynamic generation of lattice primitives [70].

## Figures and Tables

**Algorithm 1:**
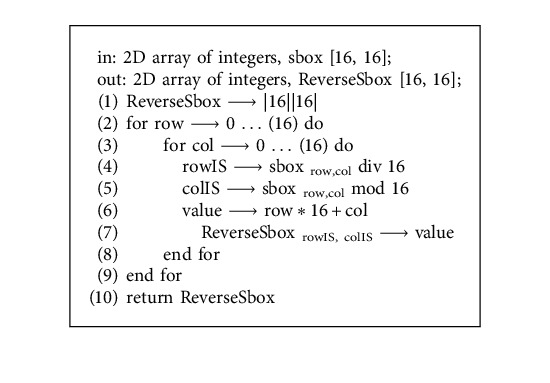
Reverse S-box (S-box).

**Table 1:** NIST statistical tests of SP-800-22.

Type of test	*P*-value	
Frequency test (monobit)	0.64785502	
Frequency test within a block	0.673576240	
Run test	0.170731649	
Longest run of ones in a block	0.875317043	
Binary matrix rank	0.285809935	
Discrete Fourier transform (spectral)	0.465626931	
Nonoverlapping template matching	0.879441943	
Cumulative sums (reverse)	0.896802069	
Cumulative sums (forward)	0.631657291	
Overlapping template matching	0.687280196	
Serial test	0.625578760	
Linear complexity	0.185625430	

Random excursions variant		
State	Chi-squared	*P*-value
−4	2.693559056	0.747103374
−3	4.472941176	0.483511959
−2	2.645606391	0.754424291
−1	8.647058824	0.123997312
1	12.29411765	0.030972537
2	1.730573711	0.885025702
3	3.344094118	0.647097954
4	3.152387486	0.676505457

Random excursions variant test		
State	Counts	*P*-value
−9	2	0.532681604
−8	5	0.595163147
−7	7	0.634322683
−6	7	0.605094946
−5	7	0.567551017
−4	6	0.475830847
−3	5	0.357385716
−2	8	0.372857936
−1	9	0.170066961
1	21	0.492716677
2	16	0.921126555
3	17	1
4	16	0.948317021
5	17	1
6	27	0.605094946
7	39	0.295361031
8	46	0.19909242
9	50	0.169870808

**Table 2:** Proposed confusion component-1.

94	133	206	66	120	92	68	118	187	114	56	167	243	93	75	143
209	64	67	36	202	151	211	57	233	162	109	21	223	150	208	161
11	203	195	180	165	37	215	157	63	28	212	78	61	213	122	72
108	231	121	90	74	250	190	8	105	31	155	216	16	160	136	185
32	7	6	152	127	25	59	44	163	49	39	198	166	81	175	159
83	60	10	13	148	204	251	3	239	69	42	123	135	228	181	17
249	196	54	230	80	189	222	244	255	110	85	176	179	182	154	221
170	19	174	15	132	43	0	86	245	177	113	234	58	142	197	207
34	12	73	146	254	134	76	124	27	218	130	2	38	186	5	252
191	242	201	219	126	106	139	156	119	115	226	103	168	45	224	220
48	210	241	140	178	173	172	138	4	248	41	227	97	89	128	40
164	30	192	141	70	235	9	77	232	125	246	199	26	200	65	253
55	184	35	238	100	101	107	1	145	102	104	82	47	112	129	144
14	205	99	169	23	194	91	53	247	217	84	98	193	171	225	240
62	236	33	116	87	79	18	183	131	22	229	20	52	214	111	88
51	46	158	96	237	149	95	188	29	153	117	71	24	147	137	50

**Table 3:** Inverse of confusion component-1.

118	199	139	87	168	142	66	65	55	182	82	32	129	83	208	115
60	95	230	113	235	27	233	212	252	69	188	136	41	248	177	57
64	226	128	194	19	37	140	74	175	170	90	117	71	157	241	204
160	73	255	240	236	215	98	192	10	23	124	70	81	44	224	40
17	190	3	18	6	89	180	251	47	130	52	14	134	183	43	229
100	77	203	80	218	106	119	228	239	173	51	214	5	13	0	246
243	172	219	210	196	197	201	155	202	56	149	198	48	26	105	238
205	122	9	153	227	250	7	152	4	50	46	91	135	185	148	68
174	206	138	232	116	1	133	92	62	254	167	150	163	179	125	15
207	200	131	253	84	245	29	21	67	249	110	58	151	39	242	79
61	31	25	72	176	36	76	11	156	211	112	221	166	165	114	78
107	121	164	108	35	94	109	231	193	63	141	8	247	101	54	144
178	220	213	34	97	126	75	187	189	146	20	33	85	209	2	127
30	16	161	22	42	45	237	38	59	217	137	147	159	111	102	28
158	222	154	171	93	234	99	49	184	24	123	181	225	244	195	88
223	162	145	12	103	120	186	216	169	96	53	86	143	191	132	104

**Table 4:** Nonlinearity of state-of-the-art techniques.

State-of-the-art confusion components	Nonlinearity score gained
[99], 2021	106.25
[101], 2021	106.5
[103], 2021	102.25
[105], 2020	106.5
[107], 2020	106.87
[109], 2020	104.25
[111], 2020	102.50
[113], 2020	106.25
[115], 2020	105.5
[117], 2021	106.75
[114], 2020	103.5
[118], 2020	106.5
[119], 2020	106.3
[121], 2021	104.0
[122], 2021	108.5
[100], 2021	109.75
[102], 2021	106.5
[104], 2021	105.5
[106], 2021	107.0
[108], 2020	105.25
[110], 2020	100.5
[112], 2020	104.0
[114], 2020	103.5
[116], 2020	105.0
[118], 2020	106.5
[111], 2020	102.5
[109], 2020	104.25
[120], 2020	101.75
[121], 2021	104.0
[123], 2021	105.25

**Table 5:** SAC of confusion component-1.

0.453125	0.500000	0.500000	0.531250	0.515625	0.500000	0.484375	0.500000
0.453125	0.562500	0.515625	0.515625	0.500000	0.468750	0.484375	0.453125
0.531250	0.515625	0.515625	0.468750	0.515625	0.500000	0.500000	0.515625
0.515625	0.468750	0.500000	0.468750	0.500000	0.500000	0.531250	0.515625
0.546875	0.515625	0.500000	0.468750	0.468750	0.546875	0.500000	0.453125
0.531250	0.515625	0.484375	0.578125	0.468750	0.515625	0.546875	0.468750
0.437500	0.515625	0.468750	0.484375	0.515625	0.500000	0.515625	0.484375
0.500000	0.406250	0.484375	0.515625	0.484375	0.500000	0.500000	0.500000

**Table 6:** SAC of BIC.

—	0.490234	0.505859	0.501953	0.513672	0.509766	0.507812	0.498047
0.490234	—	0.503906	0.513672	0.486328	0.494141	0.488281	0.480469
0.505859	0.503906	—	0.488281	0.503906	0.513672	0.513672	0.527344
0.501953	0.513672	0.488281	—	0.507812	0.490234	0.503906	0.513672
0.513672	0.486328	0.503906	0.507812	—	0.513672	0.480469	0.501953
0.509766	0.494141	0.513672	0.490234	0.513672	—	0.474609	0.470703
0.507812	0.488281	0.513672	0.503906	0.480469	0.474609	—	0.531250
0.498047	0.480469	0.527344	0.513672	0.501953	0.470703	0.531250	—

**Table 7:** DP of the confusion component-1.

.00000	.02344	.03125	.02344	.02344	.03125	.02344	.02344	.03125	.02344	.02344	.02344	.02344	.02344	.02344	.02344
.02344	.03125	.02344	.03125	.02344	.02344	.02344	.02344	.02344	.02344	.02344	.03125	.03125	.03125	.03125	.03125
.02344	.02344	.02344	.02344	.02344	.02344	.03125	.02344	.03125	.02344	.02344	.01562	.03125	.02344	.02344	.01562
.02344	.02344	.02344	.02344	.02344	.03125	.03125	.03125	.02344	.03125	.03125	.02344	.02344	.02344	.02344	.03125
.02344	.03125	.03125	.03125	.02344	.02344	.02344	.02344	.02344	.03125	.02344	.03125	.02344	.02344	.02344	.03125
.02344	.03906	.03125	.02344	.02344	.03125	.03125	.02344	.02344	.03125	.02344	.02344	.03125	.02344	.03125	.03125
.03125	.03125	.02344	.02344	.02344	.03125	.03906	.02344	.03125	.02344	.03125	.02344	.03125	.02344	.04687	.03125
.02344	.03125	.02344	.02344	.02344	.02344	.02344	.02344	.03125	.03125	.03125	.02344	.02344	.03125	.01562	.02344
.03125	.02344	.02344	.02344	.02344	.02344	.02344	.02344	.02344	.02344	.01562	.02344	.02344	.02344	.03125	.02344
.02344	.02344	.02344	.02344	.02344	.02344	.02344	.03125	.02344	.02344	.03125	.02344	.02344	.03125	.02344	.02344
.02344	.03125	.03125	.02344	.03125	.02344	.03125	.02344	.03125	.02344	.03125	.02344	.02344	.02344	.02344	.03125
.02344	.02344	.01562	.02344	.02344	.03125	.02344	.02344	.02344	.02344	.03125	.02344	.02344	.03125	.01562	.03125
.02344	.02344	.02344	.02344	.02344	.02344	.02344	.02344	.03906	.03125	.03125	.03906	.03906	.03125	.02344	.02344
.03125	.02344	.02344	.01562	.02344	.03125	.02344	.02344	.02344	.02344	.02344	.03906	.03125	.03125	.02344	.02344
.02344	.015625	.02344	.02344	.03125	.03125	.03125	.02344	.02344	.02344	.02344	.01562	.02344	.03125	.02344	.02344
.03125	.02344	.03125	.02344	.02344	.02344	.03906	.03125	.02344	.02344	.03125	.02344	.02344	.02344	.03906	.03125

**Table 8:** NPC and UACI.

Images	Location	NPCR	UACI
		Proposed	Proposed
Lena	R	99.6221	33.5514
	G	99.6127	33.5158
	B	99.5517	33.5212

Pepper	R	99.6231	33.4525
	G	99.6462	33.4642
	B	99.6652	33.4935

Nature	R	99.5925	33.6789
	G	99.6186	33.4987
	B	99.6245	33.6506

Bird	R	99.6621	33.4065
	G	99.6651	32.9154
	B	99.6266	32.9365

Baboon	R	99.6578	33.6534
	G	99.6256	33.6385
	B	99.6344	33.7265

Grapes	R	99.6231	33.7596
	G	99.6652	32.7821
	B	99.6632	33.5063

Sparrow	R	99.6551	33.4798
	G	99.6225	33.4125
	B	99.6432	32.9098

Butterfly	R	99.6591	33.5215
	G	99.6652	32.9952
	B	99.6063	33.0563

**Table 9:** Correlation analysis of the adjacent pixels.

Images	Location	Horizontal		Vertical		Diagonal	
		Plain	Encrypted	Plain	Encrypted	Plain	Encrypted
Lena	R	.9302	−.000005	.9806	.00011	.9306	.000071
	G	.9426	−.000462	.9752	−.00005	.9360	−.000051
	B	.9061	.000012	.9503	.00078	.8803	.000077

Pepper	R	.9252	.000021	.9303	.00026	.8745	.000048
	G	.9566	−.000295	.9806	−.00008	.9363	−.000065
	B	.9312	.000212	.9308	.00015	.8896	.00023

Nature	R	.9472	−.000012	.9512	.00015	.9101	−.000069
	G	.8833	.000352	.9313	−.00012	.8693	.000019
	B	.9702	.000009	.9708	−.00082	.9513	.000038

Bird	R	.9806	.000021	.9705	.00010	.9596	−.000007
	G	.9612	−.000005	.9603	.00006	.9298	.000201
	B	.9633	−.000511	.9512	.00007	.9319	−.000039

Baboon	R	.9659	−.00008	.9519	−00006	.9127	.000047
	G	.9559	.000615	.9201	−.000031	.8539	−.00078
	B	.9313	−.000018	.9499	−.00002	.9206	.000071

Grapes	R	.9836	.000051	.9826	.00005	.9568	.000068
	G	.9852	.000005	.9756	−.000031	.9627	−.000064
	B	.9788	−.000047	.9702	.00003	.9608	−0.00051

Sparrow	R	.8866	.000057	.9236	−.00004	.9906	−.000043
	G	.9503	−.000049	.8352	.00008	.9804	.000059
	B	.9306	−.000008	.7952	−.00070	.9402	.000021

Butterfly	R	.9512	−.000048	.9800	−0.0006	.8845	−.000034
	G	.8999	−.000007	.8306	.00021	.9269	.000062
	B	.8802	−.000008	.7789	.00056	.8417	.000081

## Data Availability

The datasets “EEG data from basic sensory task in Schizophrenia,” which analyzed during the current study are available in the Kaggle repository at https://www.kaggle.com/datasets/broach/button-tone-sz.

## Supplementary Materials

Supplementary Materials Figure S1: DIFFERENCE_FUSION() algorithm. Figure S2: D2DMG() algorithm. Figure S3: CONFUSIONVALUES_GENERATOR() algorithm. Figure S4: DCCG() algorithm. Table S1: confusion components. .
