# Supplementary material for: Human Psychological Disorder towards Cryptography: True Random Number Generator from EEG of Schizophrenics and Its Application in Block Encryption's Substitution Box
Source: Comput Intell Neurosci. 2022 Jun 21;2022:2532497. doi: 10.1155/2022/2532497 (PMC9239775; doi:10.1155/2022/2532497)
Supplement: Supplementary Materials — Figure S1: DIFFERENCE_FUSION() algorithm. Figure S2: D2DMG() algorithm. Figure S3: CONFUSIONVALUES_GENERATOR() algorithm. Figure S4: DCCG() algorithm. Table S1: confusion components. . [file 2532497.f1.pdf]

Figure S1

DIFFERENCE_FUSION (N, channel)

Input: N
Output: FusedArray
local: LZ1, LZ2, LZ3, LZ4, NW, NE, SW, SE, Z, GZ1, GZ2, GZ3, GZ4

```
for i ← 0 … ( N - 1) do
    LZ1 ← channel_i
    LZ2 ← channel_i+1
    LZ3 ← channel_i+2
    LZ4 ← channel_i+3
    for j ← 0 … ( | LZ1 | - 3 ) do
        GZ1 ← subSting ( LZ1, j,  j+4)
        GZ2 ← substring ( LZ2, j,  j+4 )
        GZ3 ← substring ( LZ3, j,  j+4 )
        GZ4 ← substring ( LZ4, j,  j+4 )
        if  GZ1_0 = 0  then
            NW ←subString(GZ1,0,1)+subString(GZ2,0,1)+subString(GZ1,1,2)+subString(GZ2,1,2)
        else
            NW ← subString(GZ2,0,1)+subString(GZ1,0,1) +sub(GZ2,1,2)+sub(GZ1,1,2)
        end if
        if  GZ3_0 = 0  then
            NE ← subString(GZ3,0,1)+subString(GZ4,0,1)+subString(GZ3,1,2)+subString(GZ4,1,2)
        else
            NE ← subString(GZ4,0,1)+subString(GZ3,0,1)+subString(GZ4,1,2)+subString(GZ3,1,2)
        end if
        if  GZ1_2 = 0  then
            SW ←subString(GZ1,2,3)+subString(GZ2,2,3)+subString(GZ1,3,4)+subString(GZ2,3,4)
        else
            SW ←subString(GZ2,2,3)+subString(GZ1,2,3)+subString(GZ2,3,4)+subString(GZ1,3,4)
        end if
        if  GZ3_2 = 0  then
            SE ← subString(GZ3,2,3) +subString(GZ4,2,3)+subString(GZ3,3,4)+subString(GZ4,3,4)
        else
            SE ← subString(GZ4,2,3)+subString(GZ3,2,3)+subString(GZ4,3,4)+ subString(GZ3,3,4)
        end if
        if  NW_0 = 0 then
            Z ← NW+NE+SW+SE
        else
            Z ← NE + NW + SE + SW
        end if
        FusedArray ← FusedArray + Z
        j ← j+3
    end for
    i ← i+3
end for
return FusedArray
```

Figure S2

D2DMG (FusedArray)

```
in: FusedArray
out: 2D_Map [ NMaps ] [ 4 ] [ 4 ]
local: 1D integer array frequency [ ]
| Frequency | ← | FusedArray | div 3
k ← 0
for i ←0 …( | Frequency |-1 ) do
        String3bit ← substring ( FusedArray, k ,k+3)
        Frequency_i ← String3bit.count ( 0 )
        k ← k+3
end for
Frequency ← UniqueValues ( Frequency )
y ← 0
for i ←0 … (256) do
        for row ← 0 … (4) do
                for col ← 0 …(4) do
                        2D_Map_{i, row, col} ← Frequency.get ( y )
                        y ← y+1
                end for
        end for
end for
return 2D_Map
```

Figure S3

ConfusionValuesGenerator (VectorA,VectorB, RListA, RListB, 2D_Map, Map_Index)

```
Input: VectorA[ ], VectorB[ ], RListtA[ ], RListB [ ], 2D_Map[ NMaps ][4][4], MapIndex
Output: mapIndex, SboxValuesArray [ ]
SboxValuesArray ← list( )
outputIndex ← 0
Length ← | VectorA |
countIndex2 ←  0, countIndex1 ← 0, countIndex0 ← 0
mapIndex ← MapIndex
for k ← 0 … ( Length ) do
    vAvB ← list( )
    rArB ← list( )
    for i ← 0 … (4) do
        vAvBAdd ( VectorA_i)
        rArBAdd( RListA_i)
    end for
    for i ← 0 … (4) do
        vAvBAadd ( VectorB_i )
        rArBAadd ( RListB_i )
    end for
    for i ← k … ( k + 8 ) do
        value1 ← RListA_i
        value2 ← RListB_i
        outputIndex ← 2D_Map_{mapIndex, value1,value2}
        if outputIndex = = 2 then
            for j←countIndex2 … (8) do
                countIndex2 ← countIndex2 + 1
            if outputIndex = = rArB_j then
                SboxValuesArrayAdd ( vAvB_j )
                break
            end if
            end for
        end if
        if outputIndex = = 1 then
            for j←countIndex1 … (8) do
                countIndex1←  countIndex1 + 1
            if outputIndex = = rArB_j then
                SboxValuesArrayAdd ( vAvB_j )
                break
            end if
            end for
        end if
        if outputIndex = = 0 then
            for j ← countIndex0 … (8) do
                countIndex0 ← countIndex0 + 1
            if outputIndex = = rArB_j then
                outputAdd ( vAvB_j )
                break
            end if
            end for
        end if
    end for
    mapIndex ← mapIndex + 1
    k ← k+7
end for
return SboxValuesArray
```

Figure S4

DynamicConfusionComponentGenerator(SboxValuesArray)

```
in: SboxValuesArray [ ]
out: DynamicSboxArray [ ]
for i ← 0 … ( | SboxValuesArray | - 256 ) do
        for j ← i … ( | SboxValuesArray  |- 1 ) do
                if  ( !checkNumberExists ( uniqueNum, SboxValuesArray_j ) ) then
                uniqueNum ← uniqueNum + SboxValuesArray_j + " # "
                end if
        end for
        currentSbox ← uniqueNum.split (" # ")
        if  | currentSbox | <  256 then
        break
        end if
        for j ← 0 … ( 256 ) do
        sbox ← StringToInt ( currentSbox_j )
        end for
        DynamicSboxArray.append ( sbox )
end for
return DynamicSboxArray
```

Table S1

Shannon's Confusion Component-2

213	169	228	99	157	168	107	233	183	165	117	247	187	48	34	16
167	196	120	166	217	33	5	28	44	58	67	45	20	161	37	230
179	239	134	82	90	72	30	234	49	59	180	159	88	138	85	35
137	70	77	10	79	57	94	63	129	175	29	111	243	204	246	71
17	128	194	203	145	149	42	116	176	197	26	181	226	214	227	225
23	78	8	9	83	252	140	219	1	105	84	95	170	174	7	155
91	190	158	87	198	163	36	118	96	209	210	104	162	132	186	98
201	244	61	141	220	223	55	81	178	125	249	130	142	199	241	235
193	191	164	188	200	108	52	208	206	146	11	4	148	64	73	172
152	216	221	3	92	215	2	131	245	110	51	184	74	41	124	173
122	103	229	43	238	89	182	192	222	248	143	24	240	237	202	189
32	47	147	62	251	12	13	113	114	160	250	50	31	76	46	39
19	69	212	139	54	153	115	171	255	211	101	21	109	224	15	80
207	127	231	25	119	38	121	86	133	27	65	66	75	100	144	112
218	185	177	135	254	14	0	102	53	18	151	123	60	126	68	253
106	154	40	242	236	22	205	6	156	97	93	232	195	150	136	56

Shannon's Confusion Component-3

245	7	189	111	57	61	210	208	138	199	216	197	49	18	161	213
64	218	221	12	130	155	75	192	44	107	69	235	207	170	144	145
174	50	193	150	56	97	72	8	65	46	32	201	175	209	119	132
4	135	116	9	244	204	14	33	29	100	253	79	24	15	167	127
52	35	71	196	163	67	153	230	250	36	254	105	151	136	198	211
63	251	118	16	200	112	99	194	137	148	40	165	76	240	223	85
179	94	25	172	164	166	2	6	185	62	169	195	59	181	219	45
109	187	26	147	122	89	104	93	31	180	5	239	203	17	42	60
20	234	186	157	129	128	110	77	47	184	11	233	183	248	90	80
117	113	78	1	95	19	214	229	226	48	108	160	84	241	152	3
81	0	246	242	249	237	202	176	34	217	243	115	87	73	238	222
125	247	23	83	70	37	168	51	143	159	123	156	13	66	131	205
146	140	178	28	96	91	139	41	212	142	158	114	191	21	149	173
126	121	54	39	38	182	171	252	232	27	228	86	215	101	124	225
53	206	82	162	134	98	190	133	220	30	55	227	68	88	74	103
92	224	154	188	58	231	177	120	255	141	106	22	10	102	236	43

Shannon's Confusion Component-4

143	151	209	111	66	117	113	80	122	13	86	116	35	0	34	74
82	165	228	91	203	253	29	17	71	10	204	199	241	149	161	101
105	235	20	90	120	139	135	43	83	164	131	207	185	227	255	59
244	107	194	88	47	243	230	232	148	64	96	251	110	245	163	62
94	210	153	156	187	134	9	234	184	137	218	226	175	118	221	85
246	106	170	40	169	48	97	53	102	249	127	45	217	191	67	15
49	104	65	238	72	70	176	130	46	252	177	201	202	190	8	178
214	124	220	4	180	77	37	33	36	63	250	3	150	205	50	188
193	68	41	166	22	211	7	142	222	197	208	224	181	183	61	125
76	126	79	69	39	192	92	171	154	138	216	38	95	123	195	2
18	56	141	189	52	89	206	167	145	73	99	147	32	78	237	119
200	172	42	93	84	103	55	6	152	182	81	19	231	225	160	1
198	132	223	133	5	247	26	159	128	240	31	239	108	44	24	219
212	157	144	58	236	173	186	140	11	12	30	115	174	28	155	100
27	98	196	114	109	60	158	21	129	213	75	254	136	54	215	242
168	112	25	14	248	233	162	87	16	121	57	229	51	23	179	146

Shannon's Confusion Component-5

77	53	12	72	165	120	174	17	139	96	51	164	254	1	200	105
176	66	145	83	70	117	189	138	60	220	150	134	204	132	75	166
191	154	16	208	74	160	143	109	10	255	137	238	186	201	90	247
219	24	84	98	38	11	94	95	63	217	81	30	42	40	121	115
114	242	103	89	43	29	173	141	67	192	237	162	136	142	253	64
85	144	251	140	19	49	147	21	133	171	123	243	28	73	169	225
167	202	112	188	207	87	0	113	4	48	118	78	80	213	41	32
172	187	110	79	97	211	116	205	158	170	68	20	18	65	52	119
106	15	104	62	153	5	248	126	93	13	58	235	185	102	25	127
99	59	129	122	216	218	125	245	250	193	124	128	244	39	161	135
108	223	194	111	226	56	101	175	151	107	197	246	203	3	36	233
222	50	231	210	234	146	22	45	131	23	157	180	55	2	149	57
155	249	221	159	212	214	241	232	14	8	183	184	26	6	236	168
34	100	198	152	239	46	215	163	228	61	47	82	190	182	224	196
76	31	199	44	156	88	229	148	240	130	179	209	7	252	177	54
91	230	37	227	195	69	206	27	33	9	35	181	92	86	71	178

Shannon's Confusion Component-6

79	151	253	186	169	48	178	168	121	217	138	8	163	205	35	127
108	94	218	206	43	180	174	154	224	110	70	201	109	175	96	69
41	105	20	134	12	199	78	227	211	230	77	18	247	195	21	30
17	64	107	88	237	119	147	26	162	98	136	34	90	176	213	216
156	209	55	238	114	83	54	22	179	36	27	207	129	126	33	7
104	245	125	132	131	1	188	60	66	220	51	137	65	5	46	244
56	155	225	243	50	255	57	2	235	115	61	145	252	212	192	16
93	193	29	142	250	68	246	97	111	42	81	130	23	106	241	197
219	95	80	249	157	144	183	53	128	203	152	158	45	91	87	4
102	140	139	116	232	24	208	234	254	182	59	122	13	214	10	171
242	165	82	153	173	19	248	31	196	3	63	101	159	172	37	185
202	58	84	120	187	233	161	15	75	146	215	141	32	189	149	160
47	100	28	25	11	73	200	14	226	62	92	76	103	49	228	40
181	191	223	72	9	221	38	39	231	167	240	86	210	113	0	148
229	112	67	52	166	85	71	74	135	124	118	251	123	164	190	117
99	150	177	89	6	143	133	44	184	239	236	170	204	198	222	194
